# Evaluation and prediction of land ecological security in Shenzhen based on DPSIR-TOPSIS-GM(1,1) model

**DOI:** 10.1371/journal.pone.0265810

**Published:** 2022-11-15

**Authors:** Yongjie Shan, Shaokang Wei, Weili Yuan, Yuan Miao

**Affiliations:** College of Geography Science, Shanxi Normal University, Taiyuan, Shanxi, China; Northeastern University (Shenyang China), CHINA

## Abstract

Land ecological security is the core of regional coordinated economic development and land ecological security planning. In this paper, with Shenzhen as the research area, 28 evaluation indicators were selected from 5 dimensions based on the DPSIR model to construct an indicator system for land ecological security evaluation, so as to evaluate the land ecological security status in the research area from 2009 to 2019. Based on the TOPSIS evaluation model, regional levels were determined, and finally the GM (1,1) model was adopted to scientifically predict the land ecological security system of Shenzhen from 2020 to 2025. The results showed that: (1) from the perspective of the main influencing factors, the weight of 16 indicators of Shenzhen’s land ecological security exceeds 0.03, including the total output value of agriculture, forestry, animal husbandry and fishery (*D*_*5*_) and Engel coefficient (*I*_*4*_). These factors are the main factors that have led to the deterioration of land ecological security in Shenzhen in the past decade; (2) comprehensive situation analysis revealed that from 2009 to 2019, the level of land ecological security in Shenzhen exhibited an increasing trend overall, but the land ecological security in Shenzhen still needs to be greatly improved; (3) regarding various subsystems, from 2009 to 2019, except the pressure subsystem which was in a downward trend, other subsystems showed a fluctuating and upward trend; (4) after modeling and calculation using the GM (1,1) model, it was concluded that most of the indicator factors are in a slow growing trend with the social and economic development of Shenzhen, but severe land ecological problem still exists. The research result is expected to provide a reference for the stable and sustainable development of society and economy and regional land ecosystem protection.

## 1 Introduction

Land resources are the foundation of human available resources, which threaten the survival of human beings and the sustainable development of land. At present, there is no uniformly recognized definition of the source of land ecological security [1]. Land ecological security means that within a certain range, the land ecosystem provides certain services for human survival and development, and can maintain a good state where its own structure and important functions are not threatened or less threatened. Land ecological security consists of three aspects: land natural ecological security, land economic ecological security, and land social ecology, among which land natural ecological security occupies a core position, emphasizing the capability of the land ecosystem to continuously provide ecological services or guarantee for mankind. With the social and economic development, prominent land ecological problems have appeared, such as land degradation, water and soil loss, land pollution, and decline of green space functions, which have become the hidden dangers of regional land ecological security and sustainable land use [2, 3].

In terms of the research on land ecological security, scholars at home and abroad have made many attempts in the construction of evaluation indicators, indicator weighting, and model selection. The evaluation models include PSR model [4], CLUE-S model [5], etc.; evaluation methods mainly involve AHP method [6], grey correlation model [7], spatial variability coefficient [8], and BP neural network [9]; There are comprehensive index method [10] and TOPSIS method [11] in decision analysis. And applied to industry performance evaluation [12], project site selection [13], road safety performance evaluation [14]. Yu Wenbo et al. [15] built an indicator system to evaluate urban land ecological security.

In terms of the research area, Zhang Hong et al. [16] evaluated the land ecological security in Dali City, and Liu Jiao et al. [17] measured the comprehensive value of land ecological security in 129 counties (cities, districts) in Yunnan Province; Lu Guangbin et al. [18] analyzed the overall change and spatial differentiation of land ecological security in Chongqing; Wang Yang et al. [19] conducted a dynamic evaluation of the land ecological security in Zhengzhou from 2003 to 2017, and quantitatively analyzed and identified the main obstacle factors; Li Xuemei et al. [20] investigated the spatial correlation and internal heterogeneity of land ecological security among various districts in Tianjin; Zhang Tao et al. [21] predicted the comprehensive evaluation index and coupling coordination degree between urbanization development and land ecological security in Anhui Province; Hu Dongbin et al. [22] analyzed the factors affecting land ecological security in Hunan Province; Zheng Lan et al. [23] analyzed the dynamic change of land ecological security in Jiayuguan City and the primary influencing factors; Zhang Yanwen et al. [24] explored the economic development and the level of coupling and coordination between its various subsystems and land ecological security in Guangdong. From the perspective of rural revitalization planning and development, Yang Jun et al. [25] demonstrated that with the increase of construction land in China’s Liaodong Peninsula, the development of tourism can promote the economic development and growth of China’s non-urban communities; from the perspective of simulated urban landscape characteristics, Yang et al. [26] systematically demonstrated that the mixed cellular automata model of urban landscape plays an active role in revealing urban land resource planning and land use change.

As a pilot area of China ’ s reform and opening up, Shenzhen has become a national super-large city, a national economic center city and an international city for 42 years. It is the first city of all urbanization in China. Various ecological and environmental problems and land resource utilization problems have arisen in the process of rapid urbanization [27]. Xiong Jianhua used scenario analysis method to simulate the land ecological security of Shenzhen from 2006 to 2015. It was found that the land ecological security of Shenzhen was at the level of central police and heavy police, and it was predicted to be all heavy police by 2020 [28].

In summary, land ecological security is the key link that must be paid attention to in the sustainable use of land resources in the future. Shenzhen is the concentrated achievement of China ’s reform and opening up and an international and all-urbanization city. The literature on land ecological security is few and the data is outdated, which is not conducive to the accurate grasp of land ecological security in Shenzhen. To this end, the study selected Shenzhen as a typical area to use the DPSIR model to build a land ecological security evaluation index system, and use the TOPSIS evaluation model to evaluate the land ecological security and divide the regional levels. According to the GM (1,1) model, the land ecological security system of Shenzhen in 2020–2025 was predicted, which provided a theoretical basis for Shenzhen and other regions of China to carry out land space planning and new urbanization construction.

## 2 The research area

**Fig 1:**
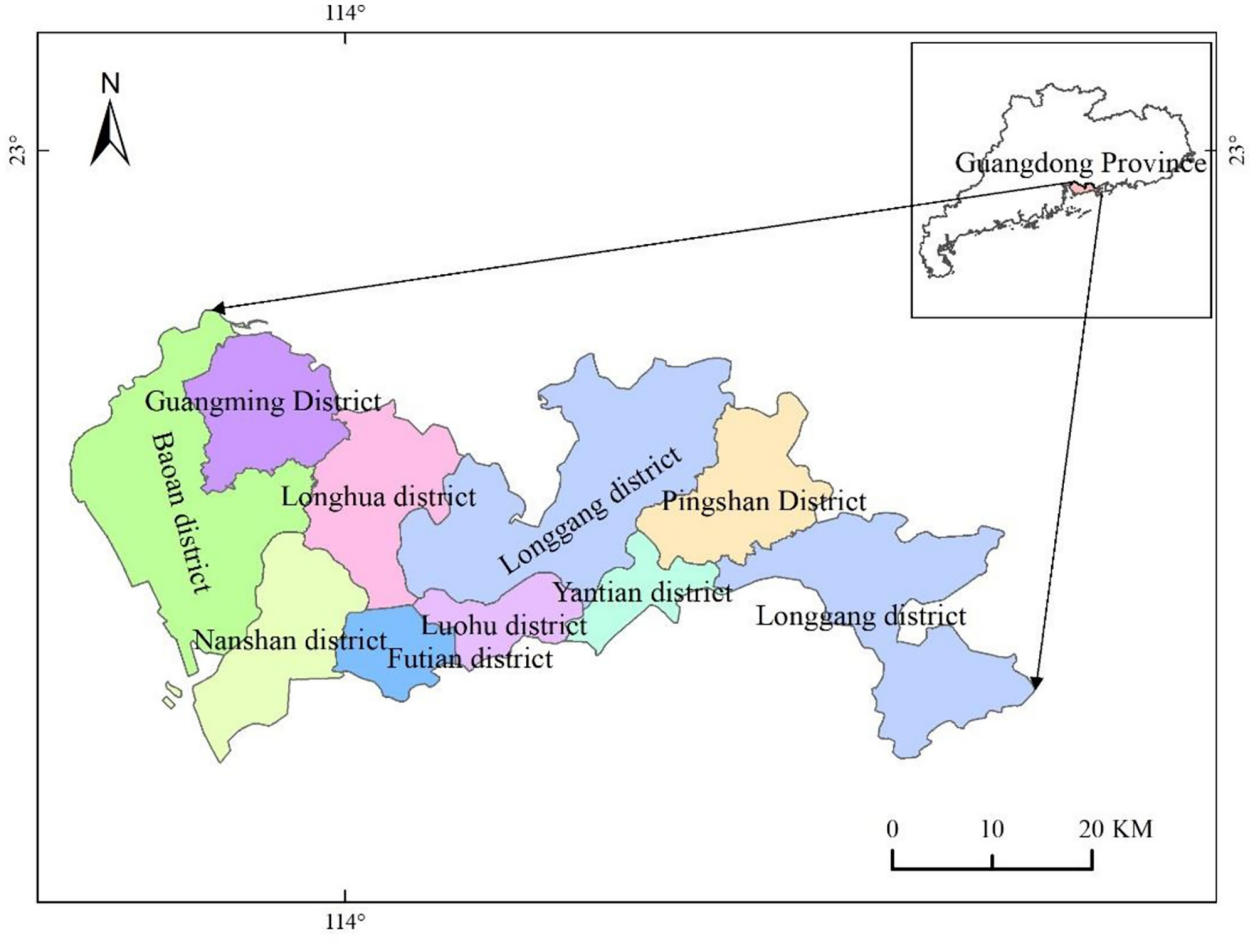
Administrative zoning map of Shenzhen city. Note: This map is from the standard map produced by GS(2019) No. 1822 of National Geographic Department of Surveying and Mapping of China, and the base map has not been modified.

Located in the southern part of Guangdong Province, 113°46’-114°37’ east longitude and 22° 27’-22°52 north latitude, Shenzhen belongs to the southern China region, with 10 districts under its jurisdiction (Fig 1). It is connected to Hong Kong by the Shenzhen River to the south. It has a subtropical monsoon climate. Shenzhen has convenient transportation by sea, land and air, ranking first in China in terms of the number of ports, the number of people leaving the country, and the traffic volume.

As of the end of 2019, Shenzhen had a permanent population of 13,438,800, including a registered population of 4.9478 million, with a population density of 6,484 people/km^2^. In 2019, it achieved a regional GDP of 2692.709 billion yuan and a per capita GDP of 203,500 yuan. It covers a total area of 1997.47 km^2^. The total sown area of crops is 122.73 km^2^, and the built-up area is 9.286 km^2^. With the social and economic development, land resources are becoming increasingly scarce and the contradiction between land supply and demand has become prominent. Therefore, the land ecological security plays a strong guiding role in social and economic development of the Pearl River Delta region and the entire Guangdong Province.

## 3 Data sources and research methods

### 3.1 Data sources

The remote sensing monitoring data of land use in this paper come from the geospatial data cloud (http://www.gscloud.cn), with a spatial resolution of 30m × 30m, Landsat8 TM remote sensing image data and DEM data were used. The data of indicators come from the “Shenzhen Statistical Yearbook”, “Guangdong Province Statistical Yearbook”, “Guangdong Statistical Bulletin” and "Statistical Yearbook of China’s Cities”, as well as the Shenzhen Economic and Social Development Statistical Bulletin. Some economic, social, and ecological environmental data come from the Guangdong Provincial Bureau of Statistics and the China Economic and Social Big Data Research Platform, as well as relevant literature.

### 3.2 Research methods

#### 3.2.1 Construction of the evaluation indicator system

DPSIR model is a new model based on PSR model and improved by European Environment Agency (EEA). It consists of five elements: driving force, pressure, state, influence and response. Each element consists of several indexes to form a complete evaluation index system. The evaluation principle of the model is that the driving force of promoting social and economic development causes great pressure on the human environment, thus changing the orderly state of the human living environment. This series of interlocking changes affect the sustainable development of human beings, the quality of the natural environment, and the order of the ecosystem, resulting in the response of all aspects of human politics, economy, environment and other measures [29]. Based on the previous research results [30–36], this paper constructs the evaluation index system of land ecological security in Shenzhen according to the principles of scientificity, practicability, operability and regionality (Table 1).

**Table 1:** Evaluation index and weight of land ecological security.

Target layer	Index layer	Index layer	Index properties	Entropy weight method *W*_*i*_	AHP method *Wj*	combination weights *Ws*
Land ecological security system	Driving force (*D*)	*D*_1_ Per capita GDP (Yuan/person)	+	0.0277	0.0313	0.0295
		*D*_2_ Growth rate of fiscal revenue (%)	-	0.0209	0.0264	0.0237
		*D*_3_ Natural population growth rate (per thousand) (‰)	-	0.0222	0.0286	0.0254
		*D*_4_ Per capita Disposable income (Yuan)	+	0.0290	0.0333	0.0312
		*D*_5_ Total output value of agriculture, Forestry, animal husbandry and fishery (ten thousand yuan)	+	0.0862	0.0662	0.0762
		*D*_*6*_ Proportion of registered population in total population (%)	-	0.0289	0.0328	0.0309
	Pressure (*P*)	*P*_1_ Population density (people /km^2^)	-	0.0372	0.0358	0.0365
		*P*_2_ GDP Growth rate (%)	+	0.0184	0.0259	0.0222
		*P*_3_ Total industrial sulfur dioxide emissions (ten thousand tons)	-	0.0233	0.0251	0.0242
		*P*_4_ Industrial solid waste Production (ten thousand tons)	-	0.0164	0.0226	0.0195
		*P*_5_ Industrial smoke and dust emission (ten thousand tons)	-	0.0131	0.0189	0.0160
		*P*_6_ Average value of Urban Road Traffic Noise (db)	-	0.0208	0.0265	0.0237
		*P*_7_ Per capita domestic electricity consumption (KWH)	-	0.0524	0.0491	0.0508
	State (*S*)	*S*_1_ Forest coverage rate (%)	+	0.0557	0.0440	0.0499
		*S*_2_ Mean value of environmental noise in Urban Area (db)	-	0.0155	0.0214	0.0185
		*S*_3_ Per capita cultivated land area (hm^2^/ person)	+	0.0357	0.0381	0.0361
		*S*_*4*_ Energy consumption per unit of GDP (KWH / ten thousand yuan)	-	0.0264	0.0313	0.0289
		*S*_*5*_ Construction Land area per capita (m^2^/ person)	-	0.0575	0.0516	0.0546
	Impact (*I*)	*I*_1_ Proportion of primary Industry (%)	+	0.0460	0.0437	0.0449
		*I*_2_ Proportion of secondary industry (%)	-	0.0464	0.0391	0.0428
		*I*_3_ Park Green area per capita (m^2^)	+	0.0169	0.0224	0.0197
		*I*_4_ Engel coefficient (%)	-	0.0750	0.0646	0.0698
		*I*_5_ Grain yield per unit Area (kg/km^2^)	+	0.0386	0.0406	0.0396
	Response (*R*)	*R*_1_ Green coverage rate of built-up area (%)	+	0.0114	0.0166	0.0140
		*R*_*2*_ Economic density (10000 yuan /km2)	+	0.0321	0.0350	0.0336
		*R*_*3*_ Public Budget Expenditure (ten thousand yuan)	+	0.0429	0.0411	0.0420
		*R*_4_ Proportion of tertiary industry (%)	+	0.0458	0.0389	0.0424
		*R*_5_ Harmless disposal rate of domestic garbage (%)	+	0.0576	0.0491	0.0534

The driving force subsystem mainly reflects the regional socio-economic development and population structure, which determine the intensity of land resource utilization and land structure. So we choose GDP per capita, fiscal revenue growth rate, per capita disposable income of residents, gross output value of agriculture, forestry, animal husbandry and fishery, natural population growth rate and the proportion of household population to reflect these two aspects. The pressure subsystem is mainly manifested as the deterioration of the ecological environment caused by land production and utilization activities, which is reflected by industrial emissions and traffic noise, population density and power consumption. The state subsystem is reflected in the changes in the number, structure and energy consumption of land, which are reflected by the per capita cultivated land area, per capita construction land area, mean value of living environment noise and energy consumption per unit of GDP. The impact subsystem shows changes in the industrial layout and increase in food production per unit area, reflected by changes in industrial restructuring, per capita green space and food production per unit area. The response subsystem reflects the change of national policy effectiveness in response to land ecological security, and selects indicators such as public budget expenditure, garbage disposal rate, built-up area greening rate, economic density and the proportion of third production.

#### 3.2.2 Normalization of evaluation indicators

Since the data unit and the positive and negative properties of each evaluation indicator are different, in order to eliminate the influence of the dimension, extreme value normalization was performed on the evaluation indicators firstly, where

The formula of positive indicators is:

$$e_{ij}=\frac{{\text{x}}_{ij}-{\text{x}}_{j\text{min}}}{{\text{x}}_{j\text{max}}-{\text{x}}_{j\text{min}}}$$ (1)

The formula of negative indicators is:

$$e_{ij}=\frac{{\text{x}}_{j\text{max}}-{\text{x}}_{ij}}{{\text{x}}_{j\text{max}}-{\text{x}}_{j\text{min}}}$$ (2)

Where *x*_*ij*_ is the actual value of each indicator, *i* = 1, …, *m* represents the number of years; *j* = 1, …, *n* represents the evaluation indicator; *x*_*jmax*_ is the maximum value of the j-th indicator, and *x*_*jmin*_ is the minimum value of the j-th indicator.

#### 3.2.3 Determination of weight

There are three methods for determining weights, namely subjective weighting method, objective weighting method and combination weighting method. The methods for weight calculation include analytic hierarchy process (AHP), coefficient of variation, Delphi model, entropy weight method, etc. In order to effectively avoid the subjective arbitrariness of the subjective weighting method [37] and the limitation of the objective weighting method that it ignores the subjective information of the decision maker [38], a combination of the entropy weight method in objective weighting method and AHP in subjective weighting method was used in this research to determine the weight of indicators. SPSS 22.0 was used to obtain the weight *W*_*j*_ of AHP, and the entropy weight method was used to obtain the weight Wi. The formula of using entropy weight method to determine weight is:

1. Calculate the entropy value of the *j*-th indicator:
$$P_{j}=-k\sum_{i=1}^{m}f_{ij}\;\text{ln}\;f_{ij}$$ (3)
  Where,and when $k=\frac{1}{\text{ln}\;m}$, $f_{ij}=\frac{e_{ij}}{\sum_{i=1}^{m}\;e_{ij}}$, *f*_*ij*_ = 0时, *f*_*ij*_ *ln f*_*ij*_ = 0.
2. Calculate the weight of the *j*-th indicator:
$$W_{j}=\frac{1-P_{j}}{\sum_{j=1}^{n}\left(1-P_{j}\right)}$$ (4)
3. Weight of the combination weighting method: This weight is the weight *Ws* of the combination weighting method formed by the combination of the weight *W*_*i*_ of entropy weight method and the weight *W*_*j*_ of AHP:
$$W_{s}=\left(1-\gamma \right)W_{i}+\gamma W_{j},0\leq \gamma \leq 1$$ (5)
  Where γ is the weighting coefficient, γ∈[0,1], the weighting coefficient γ in this paper is 0.5 according to the actual situation of the indicator system.

#### 3.2.4 TOPSIS evaluation model

The TOPSIS evaluation model obtains the final result by calculating the closeness *C*_*i*_ between the positive and negative ideal solutions and the indicators [39]. The specific steps are: the weighted normalization matrix is firstly used to obtain the optimal and worst goals, and the closeness *C*_*i*_ between the indicators and the positive and negative ideal solutions is obtained through the distance between each indicator and the optimal and worst goals.

1. Construction of weighted normalization matrix:
$$V=\left|V_{ij}\right|=W_{j}\times e_{ij}$$ (6)
  Where: *W*_*j*_ is the weight of each indicator; *e*_*ij*_ is the normalized matrix.
2. Determine the positive and negative ideal solution *V*^+^ and *V*^−^:
$$\;V^{+}=\left\{max\;v_{ij}|i=1,2,3,\cdots ,m\right\}$$ (7)
$$V^{-}=\left\{min\;v_{ij}|i=1,2,3,\cdots ,m\right\}$$ (8)
  Where: *max v*_*ij*_ and *min v*_*ij*_ are the maximum and minimum of the weighted normalized matrix respectively.
3. Calculate the distance between the indicator and the positive and negative ideal solution $S_{i}^{+}$ and $S_{i}^{-}$:
$$S_{i}^{+}=\sqrt{\sum_{j=1}^{n}{\left(V_{ij}-V_{j}^{+}\right)}^{2}}\left(i=1,2,3,\cdots ,m\right)$$ (9)
$$S_{i}^{-}=\sqrt{\sum_{j=1}^{n}{\left(V_{ij}-V_{j}^{-}\right)}^{2}}\left(i=1,2,3,\cdots ,m\right)$$ (10)
4. Calculate the closeness of the indicator C_i_:
$$C_{i}=\frac{S_{i}^{-}}{S_{i}^{+}+S_{i}^{-}}$$ (11)
  Where *C*_*i*_ is between (0–1), the smaller the value, the worse the land ecological condition in the *i*-th year, on the contrary, the larger the value of *C*_*i*_, the better the land ecological condition in the *i*-th year.

Based on the actual situation in the research area, and the existing research results [21, 28], the non-equidistant method was used to determine 5 levels of land ecological security according to the closeness *C* (Table 2):

**Table 2:** Land ecological security standard and safety level.

Security	Dangerous level	Sensitive level	Critical level	A good grade	Security level
close degree C_*i*_	(0, 0.3]	(0.3, 0.4]	(0.4, 0.6]	(0.6, 0.8]	(0.8, 1]
level	Ⅴ	Ⅳ	Ⅲ	II	Ⅰ

#### 3.2.5 Grey prediction GM(1,1) model

In order to predict the development trend of land ecological security in Shenzhen from 2020 to 2025 in a science-based manner, some available data were used to introduce the grey prediction GM (1, 1) model for modeling and prediction. The grey prediction GM(1,1) model can predict data with few data, incomplete sequence and low reliability. Without considering the distribution law or change trend, it has more scientific and accurate prediction results. This model is suitable for short-and-medium term prediction with exponential growth. In this paper, the grey prediction GM(1,1) model was employed to predict the value of *S*_*j*_^+^ and *S*_*j*_^-^ of land ecological security in Shenzhen in different years, and the mean square error ratio method was used to test the accuracy of the grey prediction GM(1,1) model. The accuracy of the model was measured by the Posterior difference ratio (*P*) and Small probability error (*C*). The evaluation criteria are as follows [31, 40] (Table 3).

**Table 3:** Grey prediction GM(1,1) model accuracy test standard.

Model accuracy grade	Posterior difference ratio (*P*)	Small probability erro (*C*)
Very well	P≤0.35	C≥0.95
Good	0.35<P≤0.50	0.80≤C<0.95
Qualified	0.50<P≤0.65	0.70≤C<0.95
Unqualified	P>0.65	C<0.7

## 4 Results and analysis

### 4.1 Analysis of the main factors affecting the land ecological security in Shenzhen

It can be seen from the results in Table 1 that weight of the following indicators of Shenzhen’s land ecological security is grater than 0.03: the total output value of agriculture, forestry, animal husbandry and fishery (*D*_*5*_), Engel coefficient (*I*_*4*_), per capita construction land area (*S*_*5*_), harmless treatment rate of domestic garbage (*R*_*5*_), per capita domestic electricity consumption (*P*_*7*_), forest coverage rate (*S*_*1*_), proportion of primary industry (*I*_*1*_), proportion of secondary industry (*I*_*2*_), proportion of tertiary industry (*R*_*4*_), public budget expenditure (*R*_*3*_), grain output per unit area (*I*_*5*_), per capita arable land area (*S*_*3*_), and per capita disposable income of residents (*D*_*4*_). These factors are the main factors that have led to the deterioration of the land ecological security in Shenzhen in the past decade. So the government needs to strengthen efforts in optimizing the industrial structure, improving the disposable income level of residents, increasing the proportion of environmental protection technology and land consolidation area, and saving and intensive land use.

### 4.2 Analysis of the comprehensive situation of land ecological security in Shenzhen

**Fig 2:**
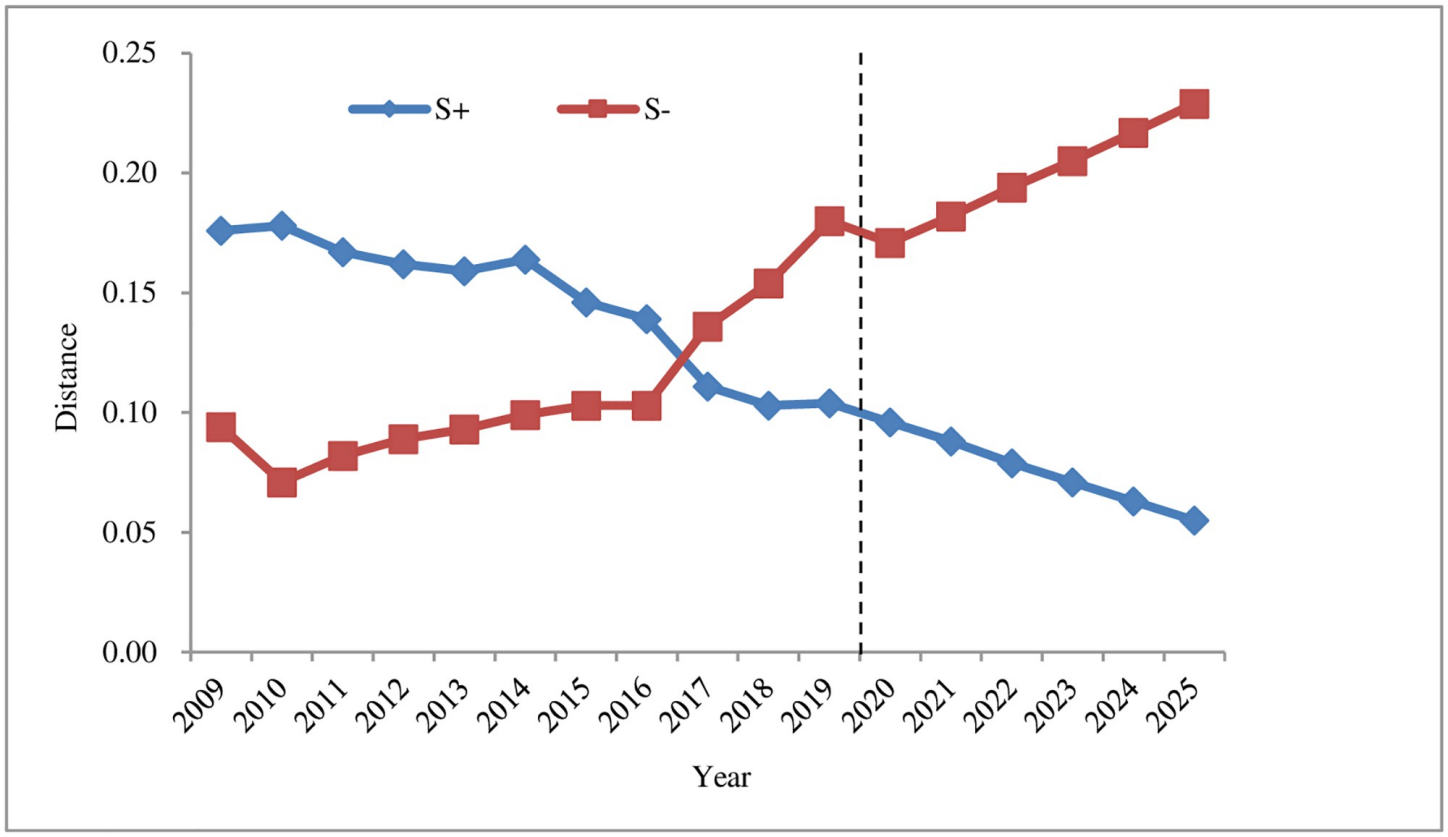
The development trend of S^+^ and S- land ecological security in Shenzhen from 2009 to 2025.

It can be seen from Fig 2 that *S*_*i*_^*+*^ showed an overall downward trend, reducing from 0.176 in 2009 to 0.104 in 2019, a decrease of 0.072, indicating that *S*_*i*_^*+*^ constantly approaches the positive ideal solution; *S*_*i*_^*-*^ exhibited an overall upward trend, rising from 0.094 in 2009 to 0.180 in 2019, an increase of 0.086, suggesting that *S*^*-*^ keeps deviating from the negative ideal solution. In 2009–2019, the closeness *C*_*i*_ firstly dropped in 2009–2010, then slowly increased in 2010–2016, and followed by a relatively rapid growth in 2016–2019. It shows that in recent years, with the continuous improvement of municipal infrastructure, the proportion of environmental indicators reaching the standard is increasing. the forest coverage, the green coverage rate in the built-up area, and the harmless treatment rate of domestic garbage in Shenzhen also exhibited a rising trend, so that the land ecological security in Shenzhen presents a trend of sustainable development.

**Fig 3:**
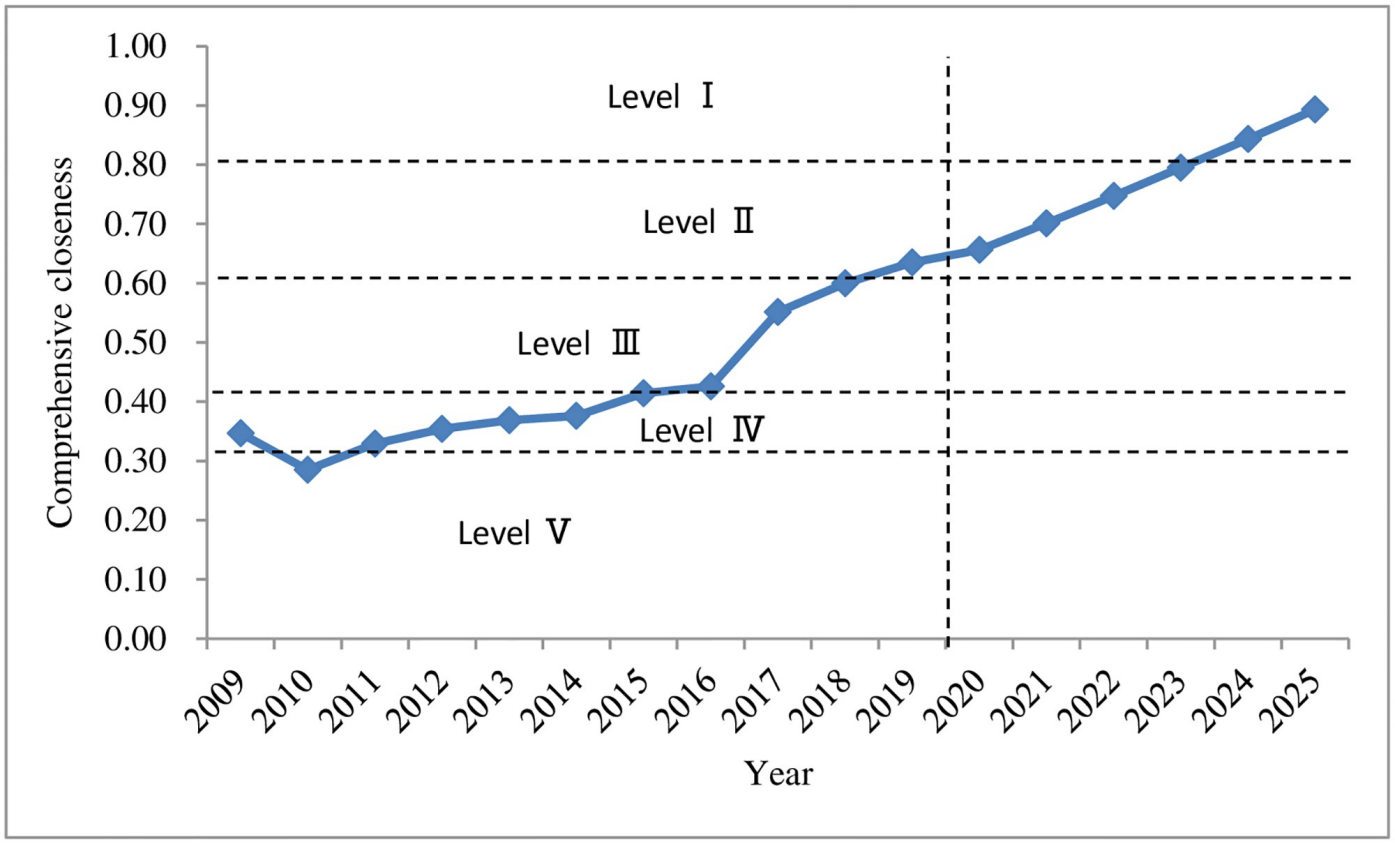
The development trend of land ecological security in Shenzhen from 2009 to 2025.

Fig 3 shows that the overall land ecological security level in Shenzhen increased from 2009 to 2019. The closeness *C*_*i*_ rose from 0.347 in 2009 to 0.635 in 2019, an increase of 0.288, changing from the sensitive level (IV) to a good level (II), indicating that the level of land ecological security has been significantly improved. However, overall, the level of land ecological security in Shenzhen is only at a good level (II), and the government still needs to devote more efforts to strictly prevent the discharge of industrial wastewater, waste gases and waste residues, the decline in the quality of arable land, and the reduction of agricultural land area, so as to improve the land ecological security level in Shenzhen.

### 4.3 Changes in various subsystems of Shenzhen’s land ecological security

**Fig 4:**
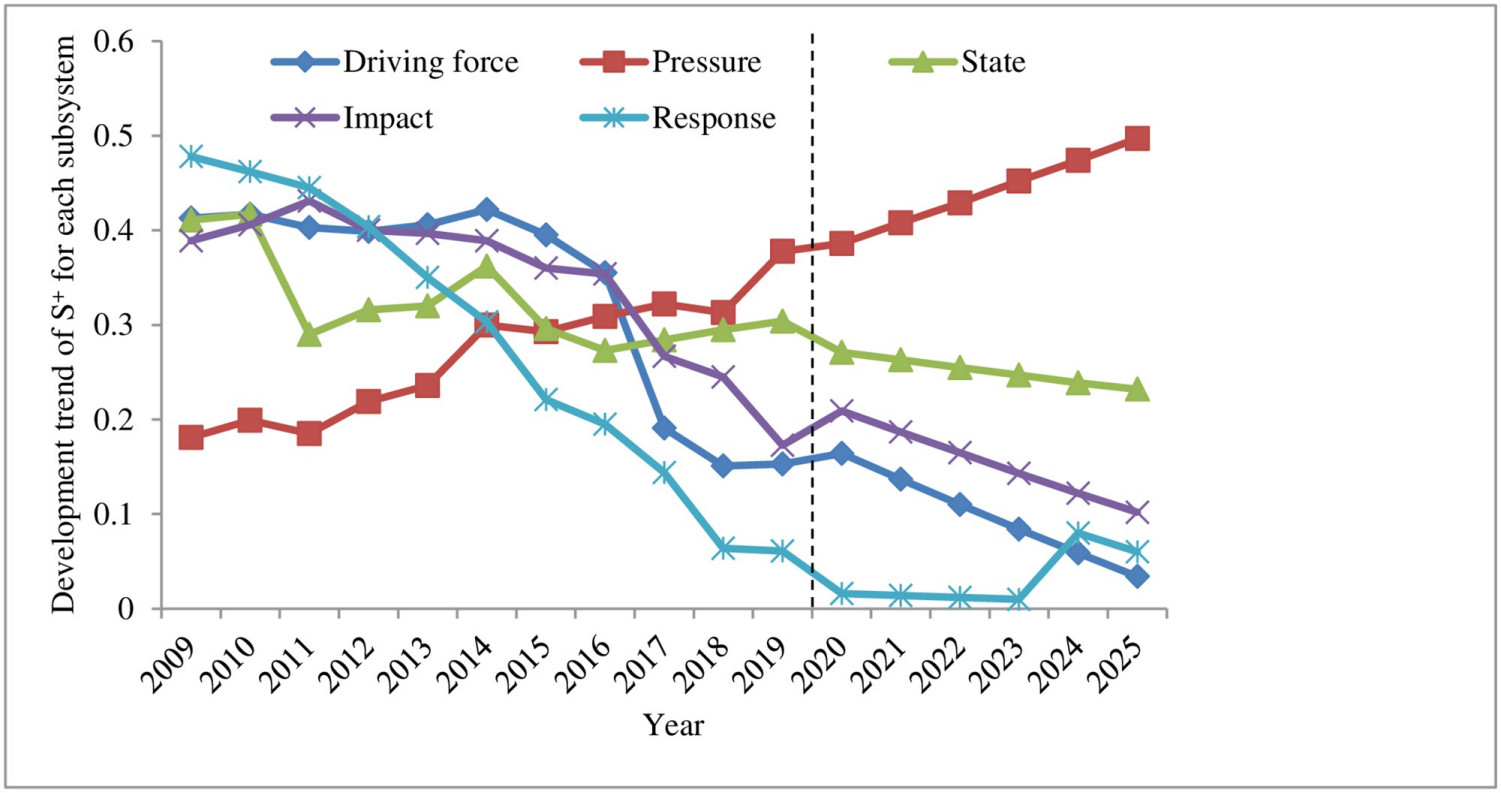
Land ecological security in Shenzhen from 2009 to 2025 subsystem S^+^ change trend.

**Fig 5:**
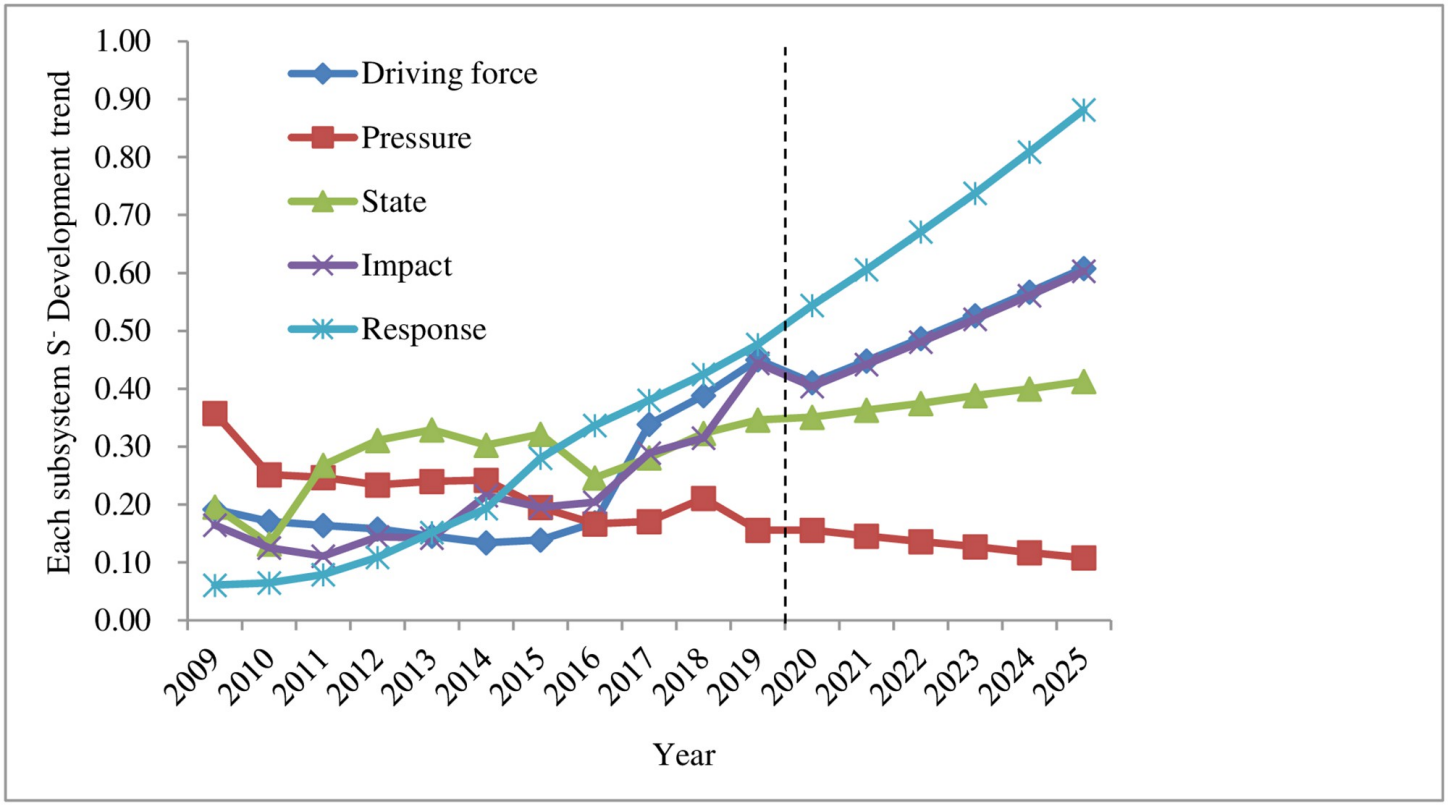
Land ecological security in Shenzhen from 2009 to 2025 subsystem S^-^change trend subsystem S^-^change trend.

**Fig 6:**
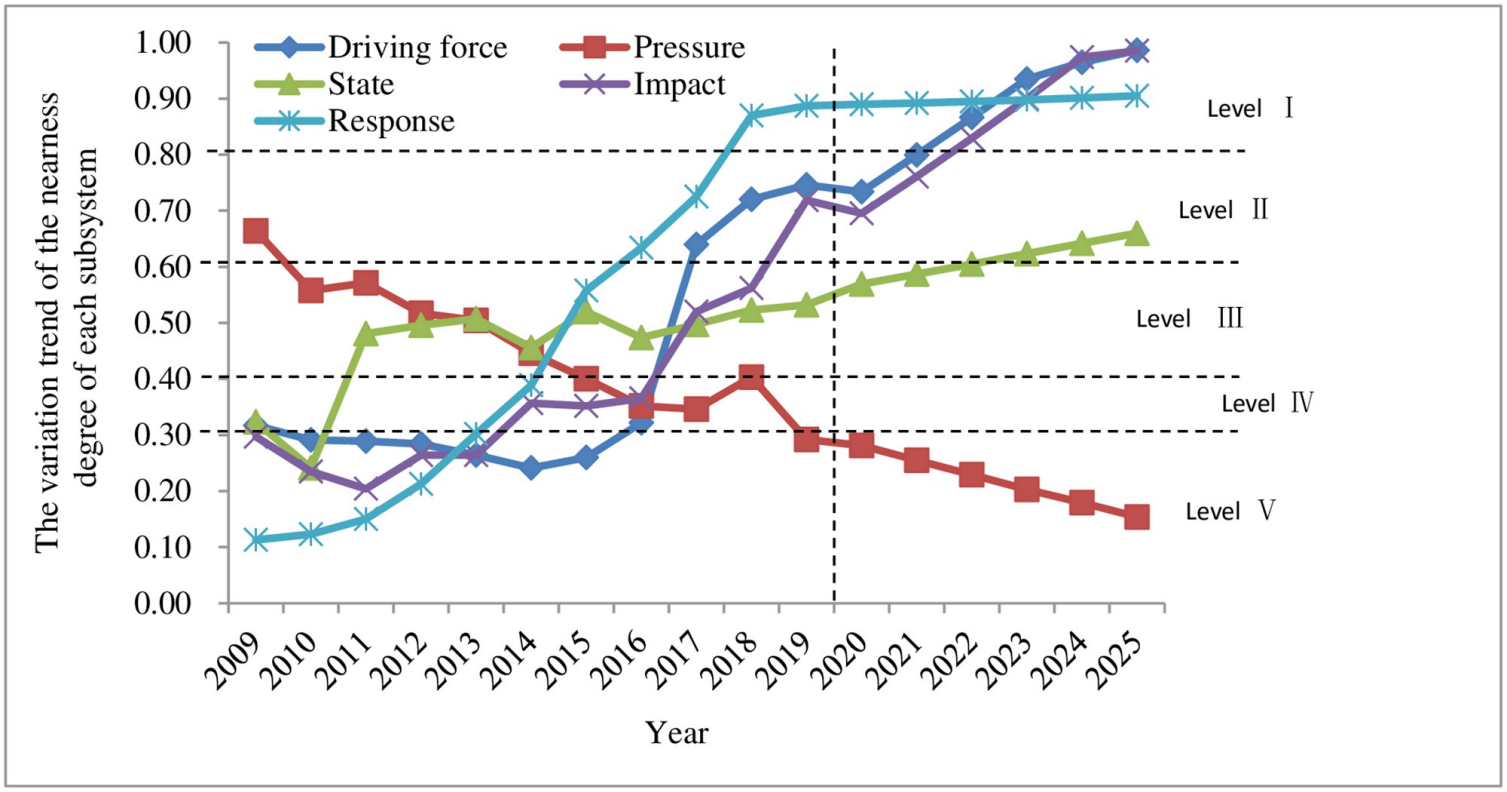
Changes in the closeness of the various subsystems of land ecological security in Shenzhen from 2009 to 2025.

1. Analysis of the driving force subsystem of land ecological security. It can be seen from Figs 4–6 that *S*_*i*_^*+*^ decreased year by year and approached the positive ideal solution, reaching the minimum value of 0.153 in 2019; *S*_*i*_^*-*^ increased year by year and deviated from the negative ideal solution, reaching the maximum value of 0.450 in 2019, indicating that the land ecological security has been improved. The closeness *C*_*i*_ increased year by year, rising from 0.317 in 2009 to 0.764 in 2019, and the security state also changed from a dangerous level (V) to a good level (II). This is closely related to the rapid economic and social development of Shenzhen in recent years. The changes in driving force indicators provide support for the land ecological security in Shenzhen.
2. Analysis of the pressure subsystem of land ecological security. Figs 4–6 reveal that *S*_*i*_^*+*^ had a fluctuating and rising trend in 2009–2019. It slightly declined in 2011, 2015, and 2018, reaching the maximum value of 0.378 in 2019; *S*_*i*_^*-*^ had an overall fluctuating and downward trend. It slightly increased in 2012–2014 and 2016–2018, reaching the minimum value of 0.156 in 2019. *S*_*i*_^*+*^ and *S*_*i*_^*-*^ are larger and smaller year by year, which makes the relative closeness *C*_*i*_ also show a decreasing trend. Shenzhen has evolved from good grade (II) in 2009 to risk grade (V) in 2019. This is the negative impact brought by the gradual improvement of infrastructure construction and the rapid economic growth in Shenzhen from 2009 to 2019, during which a great test was brought to the rapid social development of Shenzhen. Increased population density (*P*_1_), pressure on GDP growth rate (*P*_2_), industrial waste gas emissions (*P*_*3*,_ *P*_*4*,_ *P*_*5*_), average urban road traffic noise (*P*_6_), and per capita electricity consumption (*P*_7_) exerted a negative impact on the land ecology of the research area.
3. Analysis of the state subsystem of land ecological security. It can be seen from Figs 4–6 that in 2009–2019, *S*_*i*_^*+*^ firstly increased from 2009–2010, followed by a decrease and increase in 2010–2014, and dropped and rose from 2014 to 2019; *S*_*i*_^*-*^ declined rapidly from 2009 to 2010, rose sharply from 2010 to 2011, fluctuated slightly in 2011–2015, and fell slowly and then increased from 2015 to 2019, resulting in a rapid decrease and increase of relative closeness *C*_*i*_ in 2009–2011, and a fluctuating upward trend from 2011 to 2019, changing from the sensitive level (IV) in 2009 to the critical level (III) in 2019. The main reason is that during this period, the forest coverage rate (*S*_*1*_) in Shenzhen maintained a reasonable level and the average value of the urban area environmental noise (*S*_*2*_) was more reasonable. Due to the gradual improvement of the environment and the increasingly reasonable infrastructure, energy consumption per unit of GDP (*S*_*4*_) and other state indicators approach the standards of land ecological security. However, under the negative impact of the pressure subsystem, the changes in some indicators do not significantly affect the land ecosystem security in the region, such as the per capita arable land area (*S*_*3*_) and forest coverage (*S*_*1*_) indicators. In summary, the rapid expansion of construction land should not be achieved at the expense of arable land. Measures such as tree planting and afforestation and expanding the green area of built-up area to increase the green area coverage in Shenzhen are particularly important.
4. Analysis of the influence subsystem of land ecological security. It can be seen from Figs 4–6 that *S*_*i*_^*+*^ presented an overall gradual decreasing trend from 2009 to 2019, and *S*_*i*_^*-*^ exhibited a slight decline first and then fluctuated upwards overall during 2009–2018; as a result, the relative closeness *C*_*i*_ was at a dangerous level (Ⅴ) from 2009 to 2013, growing fast from 2014 to 2016, reaching a sensitive level (IV). After that, it showed a rapid growth from 2017 to 2018, arriving at a critical level (Ⅲ), and increased rapidly in 2019, standing at a good level (II). Analyzed from the various indicators affecting the subsystem, the Engel coefficient (*I*_*4*_) has been declining year by year, the proportion of the primary industry (*I*_*1*_) and the proportion of the secondary industry (*I*_*2*_) have become more reasonable, reflecting the continuous improvement of the people’s living standards and the industrial structure in Shenzhen. However, the per capita park green area (*I*_*3*_) and the grain output per unit area (*I*_*5*_) both reduced slightly, indicating that the rapid increase in construction land has led to varying degrees of reduction in the area of green and arable land, making it particularly important to increase the yield of grain. Therefore, when expanding the area of construction land, the government and other relevant departments should increase the proportion of inefficient and idle land use. Green area and arable land need to be preserved. In addition, measures such as increasing the yield of arable land should not be ignored.
5. Analysis of the response subsystem of land ecological security. It can be seen from Figs 4–6 that *S*_*i*_^*+*^ had a gradual downward trend during 2009–2011, reaching the minimum of 0.061 in 2019; *S*_*i*_^*-*^ showed a gradual upward trend in 2009–2011, reaching a maximum of 0.477 in 2019; therefore, the relative closeness *C*_*i*_ gradually increased from 2009 to 2019, reaching the maximum value of 0.887 in 2019, changing from the dangerous level (Ⅴ) in 2009 to the safety level (Ⅰ) in 2019. Judging from the specific indicators, the green coverage rate (*R*_*1*_) of built-up area was 45% from 2009 to 2018, and dropped by two percentage points to 43% in 2019. Relevant departments should pay attention to it; economic density (*R*_*2*_), public budget expenditure (*R*_*3*_), the proportion of the tertiary industry (*R*_*4*_), and the harmless treatment of domestic garbage (*R*_*5*_) all increased, suggesting that the economic density and public budget expenditure of Shenzhen increase with the social and economic development. The increase in the proportion of the tertiary industry indicates that the industrial structure has become more reasonable. The quantity and quality of service industries are important indicators for the development of an international metropolis in the future. The harmless treatment rate of domestic garbage is related to the environmental quality and the sound development of the land ecology. The improvement of these indicators plays a key role in strengthening Shenzhen’s land ecological security response subsystem.

### 4.4 Prediction of land ecological security in Shenzhen

Through the comprehensive evaluation of land ecological security in Shenzhen and the evaluation results of each subsystem, it can be concluded that the land ecological security in Shenzhen is gradually improving. However, the land ecological security status of each subsystem still fluctuates greatly. The uneven development of ecological security of some subsystems has led to a low level of land ecological security. In order to scientifically and reasonably estimate Shenzhen’s land ecological security from 2020 to 2025, DPS software was used based on the data of indicators from 2009 to 2019, and the GM (1, 1) model in grey prediction method was selected according to Shenzhen’s situation for scientific and reasonable modeling and calculation. The positive ideal solution *S*_*i*_^*+*^, the negative ideal solution *S*_*i*_^*-*^ and the relative closeness *C*_*i*_ of the comprehensive system and subsystems of Shenzhen’s land ecological security from 2020 to 2025 were systematically and scientifically predicted. The residual data were corrected to ensure the accuracy of the poorly accurate data, so that the accuracy and usability of the model prediction were enhanced. After repeated tests (Table 4), both the small probability error *C* and the posterior difference ratio *P* are within the acceptable range of reasonable usable relative error. The overall small probability error *C* of Shenzhen’s land ecological security is less than 0.54, and the small probability error *C* of each subsystem is also less than 0.54, and the posterior difference ratio *P* is greater than 0.7, verifying that the model can be used to predict land ecological security.

**Table 4:** Shenzhen city land ecological security Si^+^, Si^-^ simulation accuracy value.

Project	*S*_*i*_^*+*^			*S*_*i*_^*-*^		
	Small probability erro *C*	Posterior difference ratio *P*	Level	Small probability erro *C*	Posterior difference ratio *P*	Level
Driving force	0.4193	0.7116	Qualified	0.5373	0.7159	Qualified
Pressure	0.2153	0.8861	Good	0.2085	0.7074	Qualified
State	0.1034	0.8619	Good	0.1045	0.8799	Good
Impact	0.2123	0.7434	Qualified	0.0906	0.9758	Very well
Response	0.3384	0.7093	Qualified	0.4383	0.7046	Qualified
Comprehensive	0.2586	0.7123	Qualified	0.2746	0.7456	Qualified

According to the predicted changes in 2020–2025 (Figs 2 and 3), it can be seen that the *S*_*i*_^*+*^ of land ecological security in Shenzhen is expected to gradually decrease from 2020 to 2025, reaching a minimum value of 0.055 in 2025; compared with the value in 2019, *S*_*i*_^*-*^ will drop in 2020, followed by a gradual increase in 2020–2025, reaching a maximum of 0.229 in 2025; the relative closeness *C*_*i*_ will have a gradual upward trend, rising from 0.656 in 2020 to 0.893 in 2025, an increase of 0.237, both of which are at the safety level (I). From (Figs 4–6), it can be concluded that regarding *S*_*i*_^*+*^, the three subsystems: driving force, state, and influence will all fluctuate and decline during 2020–2025, and the response subsystem will experience a relatively fast drop in 2020–2025, while the pressure subsystem will gradually increase from 2020 to 2025; regarding *S*_*i*_^*-*^, the response subsystem will increase rapidly from 2020 to 2025, and the driving force, state, and influence subsystems will increase slightly from 2020 to 2025. The pressure subsystem will show a slight downward trend from 2020 to 2025; the comprehensive influence of the various subsystems of *S*_*i*_^*+*^ and *S*_*i*_^*-*^ result in different changes in the relative closeness *C*_*i*_ of the subsystems. The driving force and state subsystems will both slightly increase from 2020 to 2025, arriving at the safety level (I) and the good level (II) respectively. The influence and response subsystems will experience an accelerated upward trend from 2020 to 2025, reaching a safety level (Ⅰ) in 2025. The pressure subsystem is the only indicator factor that will decline rapidly from 2020 to 2025, and will be at the dangerous level (V).

Therefore, it can be concluded that from 2020 to 2025, various subsystems and the comprehensive system *C*_*i*_ of land ecological security in Shenzhen will all increase slowly except the pressure subsystem, indicating that in the process of social and economic development of Shenzhen, most indicator factors have a slow growing trend. In recent years, relevant government departments of Shenzhen have paid great attention to strengthening ecological environment protection, hence the environmental quality has maintained a good momentum. The air indicators such as SO_2_ and NO_2_ have met the national secondary standards. The quality of drinking water is good, with indicators in line with the national water quality standards for Class V water except the high values of nitrogen and phosphorus in the river. This series of standards have promoted the level of land ecological security in Shenzhen to a certain extent.

## 5 Conclusions and discussion

Through the evaluation of land ecological security in Shenzhen, the conclusions are as follows:

1. From the perspective of the main influencing factors, the weight of the following indicators of Shenzhen’s land ecological security exceeds 0.03, including total output value of agriculture, forestry, animal husbandry and fishery (*D*_*5*_), Engel coefficient (*I*_*4*_), per capita construction land area (*S*_*5*_), harmless treatment rate of domestic garbage (*R*_*5*_), per capita electricity consumption (*P*_*7*_), forest coverage rate (*S*_*1*_), proportion of primary industry (*I*_*1*_), proportion of secondary industry (*I*_*2*_), proportion of tertiary industry (*R*_*4*_), public budget expenditure (*R*_*3*_), grain output per unit area (*I*_*5*_), per capita arable land area (*S*_*3*_), and per capita disposable income of residents (*D*_*4*_). These factors are the main factors that have led to the deterioration of land ecological security in Shenzhen in the past decade.
2. Analyzing from the perspective of comprehensive analysis, from 2009 to 2019, the level of land ecological security in Shenzhen increased overall, rising from a sensitive level (IV) to a good level (II).
3. From the perspective of various subsystems of land ecological security, during 2009–2019, the driving force subsystem and the influence subsystem exhibited a fluctuating upward trend, and both were at a good level (II) in 2019; the pressure subsystem had a downward trend as a whole, changing from a good level (II) in 2009 to the dangerous level (Ⅴ) in 2019; the state subsystem experienced a fluctuating rising trend in 2019 when it was at the critical level (Ⅲ); the response subsystem had a faster rise, reaching the safety level (Ⅰ) in 2019. It shows that the driving force of land ecological security is gradually strengthened during the study period, and its influence and response are also changed into good positive influence due to the active implementation of the relevant government departments. The change of land pressure subsystem is slowly rising, which indicates that with the rapid development of social economy and the rapid realization of urbanization, the pressure on land ecological security is obviously increasing, and with the full realization of urbanization and the continuous updating and implementation of government land policy, the pressure will gradually decrease.
4. The prediction results show that the driving force, state, response and influence subsystems of Shenzhen City show a good trend from 2020 to 2025, which is at the level of safety and above. The pressure subsystem shows a downward trend, in a dangerous level, and the land ecological security comprehensive system is still in a better security level. While maintaining the rapid development of social economy, Shenzhen needs to pay attention to the institutional construction and implementation of optimizing the industrial structure and the emission control of industrial wastes.

During 2009–2019, the land ecological security level in Shenzhen was between the dangerous level (Ⅴ) and the good level (II). In the future, Shenzhen should control the rate of population growth and promote the coordinated and sustainable development of population and land; economically, low-carbon circular economy industries and high-tech industries should be introduced to optimize the tertiary industry structure. A land ecological cycle monitoring and early warning mechanism needs to be established, and a strict land ecological security reward and punishment mechanism should be formulated to promote the innovative and high-quality development of enterprises; intensive land use can be promoted in a scientific and rational manner, and the expansion of the construction land should not be realized at the expense of cultivated land, forest land, grassland, and water area. Due to the large land area and unbalanced development between regions in Shenzhen, the regional differences in land ecological security objectively exist. The spatial-temporal difference analysis and influencing factors of land ecological security in various administrative regions of Shenzhen have become the next work direction.

## Supporting information

S1 Data (XLSX)
